# Design, synthesis and biological activity of pyrazinamide derivatives for anti-*Mycobacterium tuberculosis*

**DOI:** 10.1080/14756366.2017.1367774

**Published:** 2017-09-04

**Authors:** Shiyang Zhou, Shanbin Yang, Gangliang Huang

**Affiliations:** a College of Chemistry, Chongqing Normal University, Chongqing, China;; b University Bioactive Substance Engineering Research Center in Chongqing, Chongqing Normal University, Chongqing, China

**Keywords:** Pyrazinamide, derivatives, design, synthesis, biological activity

## Abstract

A total of 11 pyrazinamide derivatives were designed and synthesised using pyrazinamide as the lead compound, which was optimised by structural modification with alkyl chains, six-membered rings, and bioisosterism, respectively. The target compounds were synthesised using pyrazinecarboxylic acid as the starting material by acylation, amidation, and alkylation, respectively. Their structures were confirmed by ^1^H NMR, ^13^C NMR, HRESIMS, and elemental analysis, respectively. The bioactivities of derivatives were assayed using bacteriostatic experiment and minimum inhibitory concentration experiment. It was showed that the derivatives had good inhibitory effect on *Mycobacterium tuberculosis*. The biological activity of derivative **1f** was the best among all compounds, its antibacterial activity was 99.6%, and the minimum inhibitory concentration was 8.0 µg/mL.

## Introduction

**Figure 1.**
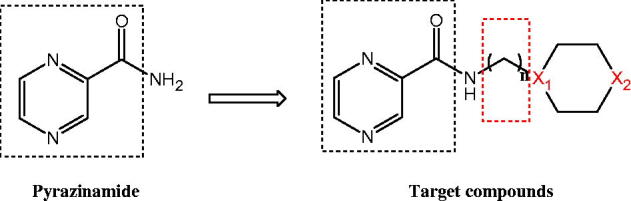
Design of pyrazinamide derivatives.

Tuberculosis (TB) is a common and fatal chronic infectious disease, and it is easy to occur in young people. Its clinical manifestations include low fever, night sweats, weakness or emaciation, and respiratory symptoms, such as cough and hemoptysis. The disease is mainly caused by *Mycobacterium tuberculosis* infection, also from *M. bovis*, *M. africanum*, *M. canetti*, *M. microti*, and other species of *M. tuberculosis* infection, but the mycobacteria do not usually infect healthy adults^1–3^. TB usually infects the lungs, brain, lymph system, central nervous system, urinary system, circulatory system, bones, joints, and even some skin can also be infected. At present, there are about 600 million people worldwide to have the Bacillus *M. tuberculosis* infection, and most infected people are in the developing world. The infection rate is the highest in Africa’s Per Capita, and most infected people have no corresponding symptoms for latent tuberculosis infection^4–6^. With the impact of environmental pollution and the spread of AIDS, the incidence of TB is increasing. The WHO announced in 1993 that TB was a global health emergency, while the termination of TB partners proposed the “global TB prevention program”. In 1882, scientists found that the pathogenic bacterium of tuberculosis for *M. tuberculosis*. Until the 50s of the last century, the researchers found drugs which could inhibit *M. tuberculosis*, and the TB infection control was effective. At present, the drugs commonly used in clinical trials for anti-tuberculosis include isoniazid, rifampicin, pyrazinamide, and ethambutol^7^. Latent TB was usually used as a single drug, while active TB was most appropriate to take several drugs at the same time for reducing the risk of drug-resistant bacteria^8^^,^^9^. In view of the great harm of TB, it is of great significance to study new anti-disease drugs. Pyrazinamide has good inhibitory effect on *M. tuberculosis* at pH 5–5.5, and the inhibition of bacteria is the strongest. The minimum inhibitory concentration of pyrazinamide in the human body is 12.5 µg/mL, and it can directly kill *M. tuberculosis in vivo* when the concentration reaches 50 µg/mL. The metabolite of pyrazinamide during antibacterial mechanism is pyrazinoic acid. When pyrazinamide gets into *M. tuberculosis* bacteria *in vivo*, the pyrazinoic acid metabolite achieves the bacteriostatic effect. In addition, it can also replace the nicotinamide to prevent dehydrogenase interference and dehydrogenation, interfere with *M. tuberculosis* on oxygen utilisation, affect the normal metabolism, and result in the death of bacteria *M. tuberculosis*. The pyrazinamide has good antibacterial effect, but there are side effects, and the main side effect is to cause liver damage. In addition, the water/fat-solubility of pyrazinamide is not good. Our research group designed and synthesised a new class of pyrazinamide compounds that achieved better bacteriostatic effect. The structure of pyrazinamide was optimised by structure modification with alkyl chains/six membered rings and bioisosterism. The design (Figure 1) of pyrazinamide derivatives was as follows.

## Experimental

### Synthetic experiments

#### Synthesis of pyrazine-2-carboxylic acid chloride **(3)**

The pyrazine carboxylic acid (12.41 g, 0.10 mol) was put into 250 mL round bottom flask, then 100 mL methylene chloride as solvent and 5 drops of *N,N*-dimethyl formamide (DMF) used as catalyst for the reaction were added. The flask was placed in the ice water bath, and a magnetic mixer was used to stir until the reaction liquid became clarified. The thionyl chloride (29 mL, 0.40 mol) was constantly dropped into the flask under stirring, and the drop speed rate and reaction temperature were controlled. After the reactants were added, the reaction lasted 8 h under refluxing. The methylene chloride and excessive thionyl chloride were removed under vacuum. The mixture was dried to get the crude product of pyrazine formyl chloride. The crude product was recrystallised with toluene, filtered, and dried, respectively. The pure pyrazine-2-carboxylic acid chloride (**3**) was obtained as the white crystal^10^.

#### A general method for synthesis of compounds ***4a*** to ***4c***

The pyrazine formyl chloride (14.25 g, 0.10 mol) was put into 250 mL round bottom flask, and 16 mL (0.20 mol) bromide methylamine and 100 mL toluene solvent were added. The reaction liquid was magnetically stirred, and the reaction lasted 8 h under refluxing. The reaction mixture was thermally filtrated and cooled. The crude product was recrystallised with toluene, filtered, and dried, respectively. The pure *N*-(1-bromine methyl) pyrazine formamide (**4a**) as white crystal was obtained. The general method was used to synthesise compounds **4b**–**4c**.

*N*-(bromomethyl)pyrazine-2-carboxamide (**4a**): white crystal; ^1^H NMR (CDCl_3_, 300 MHz): *δ* = 9.96 (1H, s, pyrazine-H), 9.38 (1H, s, –NH–), 9.10 (1H, dd, *J* = 8.0 Hz, pyrazine-H), 8.89 (1H, dd, *J* = 8.0 Hz, pyrazine-H), 4.97 (2H, s, *J* = 6.4 Hz, –CH_2_–); ^13^C NMR (CDCl_3_, 75 MHz): *δ* = 161.0 (C=O), 146.0, 145.0, 144.7, 144.6 (pyrazine-C), 45.4 (CH_2_); HRESIMS *m/z* (pos): 216.0376 C_6_H_6_N_3_OBr (calcd. 215.7230); Anal. Calcd. for C_6_H_6_N_3_OBr: C, 33.36; H, 2.80; N, 19.45; Br, 36.99. Found: C, 33.37; H, 2.81; N, 19.43; Br, 36.98%.

*N*-(2-bromoethyl)pyrazine-2-carboxamide (**4b**): white crystal; ^1^H NMR (CDCl_3_, 300 MHz): *δ* = 9.96 (1H, s, pyrazine-H), 9.10 (1H, dd, *J* =7.9 Hz, pyrazine-H), 8.98 (1H, s, –NH–), 8.89 (1H, dd, *J* = 7.9 Hz, pyrazine-H), 3.74 (4H, s, *J* = 6.3 Hz, –CH_2_–); ^13 ^C NMR (CDCl_3_, 75 MHz): *δ* = 160.7 (C=O), 146.0, 145.0, 144.7, 144.6 (pyrazine-C), 46.6, 30.9 (CH_2_CH_2_); HRESIMS *m/z* (pos): 230.0653 C_7_H_8_N_3_OBr (calcd. 229.3426); Anal. Calcd. for C_7_H_8_N_3_OBr: C, 36.54; H, 3.51; N, 18.26; Br, 34.73. Found: C, 36.55; H, 3.52; N, 18.26; Br, 34.72%.

*N*-(3-bromopropyl)pyrazine-2-carboxamide (**4c**): white crystal; ^1^H NMR (CDCl_3_, 300 MHz): *δ* = 9.96 (1H, s, pyrazine-H), 9.10 (1H, dd, *J* =7.8 Hz, pyrazine-H), 8.98 (1H, s, –NH–), 8.89 (1H, dd, *J* =7.8 Hz, pyrazine-H), 3.51 (2H, m, *J* = 6.0 Hz, –CH_2_–), 3.18 (2H, m, *J* = 6.0 Hz, –CH_2_–), 2.05 (2H, m, *J* = 6.0 Hz, –CH_2_–); ^13^C NMR (CDCl_3_, 75 MHz): *δ* = 160.7 (C=O, 146.0, 145.0, 144.7, 144.6 (pyrazine-C), 38.4, 32.6, 30.8 (CH_2_CH_2_CH_2_); HR-ESI-MS *m/z* (pos): 244.0921 C_8_H_10_N_3_OBr (calcd. 243.5438); Anal. Calcd. for C_8_H_10_N_3_OBr: C, 39.37; H, 4.13; N, 17.22; Br, 32.74. Found: C, 39.38; H, 4.14; N, 17.21; Br, 32.73%.

#### A general method for all titled derivatives **1a**–1k

*N-*(1-bromine methyl) pyrazine formamide (21.60 g, 0.10 mol) was put into 250 mL round-bottomed flask, and 21.5 mL (0.20 mol) six-membered ring compound and 100 mL toluene were added. The reaction liquid was magnetically stirred, and the reaction lasted 12 h under refluxing. The reaction mixture was filtrated and cooled. The crude product was recrystallised with toluene, filtered, and dried, respectively. The target (**1**) as white crystal was obtained.

*N*-(piperidin-1-ylmethyl)pyrazine-2-carboxamide (**1a**): white crystal; yield 86.2%; m.p. 183–185 °C; ^1^H NMR (CDCl_3_, 300 MHz): *δ* = 9.96 (1H, s, pyrazine-H), 9.38 (1H, s, –NH–), 9.10 (1H, dd, *J* = 7.8 Hz, pyrazine-H), 8.89 (1H, dd, *J* = 7.8 Hz, pyrazine-H), 4.03 (2H, s, *J* = 6.0 Hz, N–CH_2_–), 2.39 (4H, m, –CH_2_–), 1.43 (4H, m, –CH_2_–), 1.30 (2H, m, –CH_2_–); ^13 ^C NMR (CDCl_3_,75 MHz): *δ* = 161.0 (C=O), 146.6, 145.0, 144.7, 144.6 (pyrazine-C), 66.1 (CH_2_, N–CH_2_), 54.2, 25.6, 24.5 (CH_2_, CH_2_–CH_2_); HRESIMS *m/z* (pos): 220.2760 C_11_H_16_N_4_O (calcd. 220.1324); Anal. Calcd. for C_11_H_16_N_4_O: C, 59.98; H, 7.32; N, 25.44. Found: C, 59.99; H, 7.31; N, 25.43.

*N*-(piperazin-1-ylmethyl)pyrazine-2-carboxamide (**1b**): white crystal; yield 88.2%; m.p. 190–192 °C; ^1^H NMR (CDCl_3_, 300 MHz): *δ* = 9.96 (1H, s, pyrazine-H), 9.38 (1H, s, –NH–), 9.10 (1H, dd, *J* = 7.6 Hz, pyrazine-H), 8.89 (1H, dd, *J* = 7.6 Hz, pyrazine-H), 4.03 (2H, s, *J* = 5.9 Hz, N–CH_2_–), 2.65 (4H, m, –CH_2_–), 2.34 (4H, m, –CH_2_–), 1.07 (1H, s, –NH–); ^13^C NMR(CDCl_3_, 75 MHz): *δ* = 161.0 (C=O), 146.6, 145.0, 144.7, 144.6 (pyrazine-C), 66.1 (CH_2_, N–CH_2_), 54.7, 45.9 (CH_2_, CH_2_–CH_2_); HRESIMS *m/z* (pos): 221.2640 C_10_H_15_N_5_O (calcd. 221.1277); Anal. Calcd. for C_10_H_15_N_5_O: C, 54.28; H, 6.83; N, 31.65. Found: C, 54.29; H, 6.83; N, 31.64.

*N*-(thiomorpholinomethyl)pyrazine-2-carboxamide (**1c**): white crystal; yield 86.3%; m.p. 201–203 °C; ^1^H NMR (CDCl_3_, 300 MHz): *δ* = 9.96 (1H, s, pyrazine-H), 9.38 (1H, s, –NH–), 9.10 (1H, dd, *J* = 7.7 Hz, pyrazine-H), 8.89 (1H, dd, *J* = 7.7 Hz, pyrazine-H), 4.03(2H, s, *J* = 6.0 Hz, N–CH_2_–), 2.73 (4H, m, –CH_2_–), 2.54 (4H, m, –CH_2_–); ^13 ^C NMR (CDCl_3_, 75 MHz): *δ* = 161.0 (C=O), 146.6, 145.0, 144.7, 144.6 (pyrazine-C), 65.4 (CH_2_, N–CH_2_), 57.7, 28.0 (CH_2_, CH_2_–CH_2_); HRESIMS *m/z* (pos): 238.3090 C_10_H_14_N_4_OS (calcd. 238.0888); Anal. Calcd. for C_10_H_14_N_4_OS: C, 50.40; H, 5.92; N, 23.51; S, 13.45. Found: C, 50.41; H, 5.91; N, 23.50; S, 13.46%.

*N*-(2-(piperidin-1-yl)ethyl)pyrazine-2-carboxamide (**1d**): white crystal; yield 84.0%; m.p. 185–187 °C; ^1^H NMR (CDCl_3_, 300 MHz): *δ* = 9.96 (1H, s, pyrazine-H), 9.10 (1H, dd, *J* = 7.6 Hz, pyrazine-H), 8.89 (1H, dd, *J* = 7.6 Hz, pyrazine-H), 8.81 (1H, s, –NH–), 3.36 (2H, s, *J* = 5.8 Hz, N–CH_2_–), 2.46 (2H, s, –CH_2_–), 2.42 (4H, m, –CH_2_–), 1.49 (4H, m, –CH_2_–), 1.37 (2H, m, –CH_2_–); ^13^C NMR (CDCl_3_, 75 MHz): *δ* = 160.7 (C=O), 146.6, 145.0, 144.7, 144.6 (pyrazine-C), 57.2 (CH_2_, N–CH_2_), 56.8, 54.0, 25.9, 24.5 (CH_2_, CH_2_–CH_2_); HRESIMS *m/z* (pos): 234.1481 C_12_H_18_N_4_O (calcd. 234.3030); Anal. Calcd. for C_12_H_18_N_4_O: C, 61.52; H, 7.74; N, 23.91. Found: C, 61.53; H, 7.75; N, 23.90.

*N*-(2-(piperazin-1-yl)ethyl)pyrazine-2-carboxamide (**1e**): white crystal; yield 90.4%; m.p. 192–193 °C; ^1^H NMR (CDCl_3_, 300 MHz): *δ* = 9.96 (1H, s, pyrazine-H), 9.10 (1H, dd, *J* = 7.7 Hz, pyrazine-H), 8.89 (1H, dd, *J* = 7.7 Hz, pyrazine-H), 8.81 (1H, s, –NH–), 3.36 (2H, s, *J* = 5.9 Hz, N–CH_2_–), 2.65 (4H, m, –CH_2_–), 2.46 (2H, s, –CH_2_–), 2.34 (4H, m, –CH_2_–), 1.07 (1H, s, –NH_2_–); ^13^C NMR (CDCl_3_, 75 MHz): *δ* = 160.7 (C=O), 146.6, 145.0, 144.7, 144.6 (pyrazine-C), 57.3 (CH_2_, N–CH_2_), 57.2, 56.8, 53.7, 46.2 (CH_2_, CH_2_–CH_2_); HRESIMS *m/z* (pos): 235.1433 C_11_H_17_N_5_O (calcd. 235.2910); Anal. Calcd. for C_11_H_17_N_5_O: C, 56.15; H, 7.28; N, 29.77. Found: C, 56.16; H,7.27; N, 29.78.

*N*-(2-morpholinoethyl)pyrazine-2-carboxamide (**1f**): white crystal; yield 91.2%; m.p. 202–204 °C; ^1^H NMR (CDCl_3_, 300 MHz): *δ* = 9.96 (1H, s, pyrazine-H), 9.10 (1H, dd, *J* = 7.8 Hz, pyrazine-H), 8.89 (1H, dd, *J* = 7.8 Hz, pyrazine-H), 8.81 (1H, s, –NH–), 3.50 (4H, m, –CH_2_–), 3.36 (2H, s, *J* = 6.0 Hz, N–CH_2_–), 2.46 (2H, s, –CH_2_–), 2.40 (4H, m, –CH_2_–); ^13^C NMR (CDCl_3_, 75 MHz): *δ* = 160.7 (C=O), 146.6, 145.0, 144.7, 144.6 (pyrazine-C), 66.7 (CH_2_, N–CH_2_), 57.2, 55.8, 54.0 (CH_2_, CH_2_–CH_2_); HRESIMS *m/z* (pos): 236.2750 C_11_H_16_N_4_O_2_ (calcd. 236.1273); Anal. calcd for C_11_H_16_N_4_O_2:_ C, 55.92; H, 6.83; N, 23.71. Found: C, 55.91; H, 6.84; N, 23.70.

*N*-(2-thiomorpholinoethyl)pyrazine-2-carboxamide (**1g**): white crystal; yield 90.4%; m.p. 192–193 °C; ^1^H NMR (CDCl_3_, 300 MHz): *δ* = 9.96 (1H, s, pyrazine-H), 9.10 (1H, dd, *J* = 7.7 Hz, pyrazine-H), 8.89 (1H, dd, *J* = 7.7 Hz, pyrazine-H), 8.81 (1H, s, –NH–), 3.36 (2H, s, *J* = 5.9 Hz, N–CH_2_–), 2.73 (4H, m, –CH_2_–), 2.54 (4H, m, –CH_2_–), 2.46 (2H, s, –CH_2_–); ^13^C NMR (CDCl_3_, 75 MHz): *δ* = 160.7 (C=O), 146.6, 145.0, 144.7, 144.6 (pyrazine-C), 60.3 (CH_2_, N–CH_2_), 57.2, 53.3, 28.3 (CH_2_, CH_2_–CH_2_); HRESIMS *m/z* (pos): 252.3360 C_11_H_16_N_4_OS (calcd. 252.1045); Anal. Calcd. for C_11_H_16_N_4_OS: C, 52.36; H, 6.39; N, 22.20; S, 12.71. Found: C, 52.35; H, 6.40; N, 22.18; S, 12.72%.

*N*-(3-(piperidin-1-yl)propyl)pyrazine-2-carboxamide (**1h**): white crystal; yield 81.0%; m.p. 204–205 °C; ^1^H NMR (CDCl_3_, 300 MHz): *δ* = 9.96 (1H, s, pyrazine-H), 9.10 (1H, dd, *J* = 7.5 Hz, pyrazine-H), 8.98 (1H, s, –NH–), 8.89 (1H, dd, *J* = 7.5 Hz, pyrazine-H), 3.18 (2H, s, *J* = 5.8 Hz, N–CH_2_–), 2.42 (4H, m, –CH_2_–), 2.36 (2H, s, –CH_2_–), 1.73 (2H, s, –CH_2_–), 1.49 (4H, m, –CH_2_–), 1.37 (2H, m, –CH_2_–); ^13^C NMR(CDCl_3_, 75 MHz): *δ* = 160.7 (C=O), 146.6, 145.0, 144.7, 144.6 (pyrazine-C), 57.1 (CH_2_, N–CH_2_), 40.4, 39.7, 27.0, 25.9, 24.5 (CH_2_, CH_2_–CH_2_); HRESIMS *m/z* (pos): 248.3300 C_13_H_20_N_4_O (calcd. 248.1637); Anal. Calcd. for C_13_H_20_N_4_O: C, 62.88; H, 8.12; N, 22.56. Found: C, 62.89; H, 8.13; N, 22.55.

*N*-(3-(piperazin-1-yl)propyl)pyrazine-2-carboxamide (**1i**): white crystal; yield 84.6%; m.p. 210–211 °C; ^1^H NMR (CDCl_3_, 300 MHz): *δ* = 9.96 (1H, s, pyrazine-H), 9.10 (1H, dd, *J* = 7.6 Hz, pyrazine-H), 8.98 (1H, s, –NH–), 8.89 (1H, dd, *J* = 7.6 Hz, pyrazine-H), 3.18 (2H, s, *J* = 5.7 Hz, N–CH_2_–), 2.65 (4H, m, –CH_2_–), 2.36 (2H, s, –CH_2_–), 2.34 (4H, m, –CH_2_–), 1.73 (2H, s, –CH_2_–), 1.07 (2H, s, –CH_2_–); ^13^C NMR (CDCl_3_, 75 MHz): *δ* = 160.7 (C=O), 146.6, 145.0, 144.7, 144.6 (pyrazine-C), 57.6 (CH_2_, N–CH_2_), 51.6, 46.2, 39.7, 27.0 (CH_2_, CH_2_–CH_2_); HRESIMS *m/z* (pos): 249.3180 C_12_H_19_N_5_O (calcd. 249.1590); Anal. Calcd. for C_12_H_19_N_5_O: C,57.81; H, 7.68; N, 28.09. Found: C, 57.80; H, 7.69; N, 28.10.

*N*-(3-morpholinopropyl)pyrazine-2-carboxamide (**1j**): white crystal; yield 87.3%; m.p. 221–223 °C; ^1^H NMR (CDCl_3_, 300 MHz): *δ* = 9.96 (1H, s, pyrazine-H), 9.10 (1H, dd, *J* = 7.7 Hz, pyrazine-H), 8.98 (1H, s, –NH–), 8.89 (1H, dd, *J* = 7.7 Hz, pyrazine-H), 3.55 (4H, m, –CH_2_–), 3.18 (2H, s, *J* = 5.7 Hz, N–CH_2_–), 2.37 (2H, s, –CH_2_–), 2.33 (4H, m, –CH_2_–), 1.73 (2H, s, –CH_2_–); ^13^C NMR (CDCl_3_, 75 MHz): *δ* = 160.7 (C=O), 146.6, 145.0, 144.7, 144.6 (pyrazine-C), 62.9 (CH_2_, N–CH_2_), 59.4, 39.7, 27.0 (CH_2_, CH_2_–CH_2_); HRESIMS *m/z* (pos): 250.3020 C_12_H_18_N_4_O_2_ (calcd. 250.1430); Anal. Calcd. for C_12_H_18_N_4_O_2_: C, 57.58; H, 7.25; N, 22.38. Found: C, 57.59; H, 7.24; N, 22.37.

*N*-(3-thiomorpholinopropyl)pyrazine-2-carboxamide (**1k**): white crystal; yield 84.3%; m.p. 230–231 °C; ^1^H NMR (CDCl_3_, 300 MHz): *δ* = 9.96 (1H, s, pyrazine-H), 9.10 (1H, dd, *J* = 7.6 Hz, pyrazine-H), 8.98 (1H, s, –NH–), 8.89 (1H, dd, *J* = 7.6 Hz, pyrazine-H), 3.18 (2H, s, *J* = 5.6 Hz, N–CH_2_–), 2.73 (4H, m, –CH_2_–), 2.54 (4H, m, –CH_2_–), 2.36 (2H, s, –CH_2_–), 1.73 (2H, s, –CH_2_–); ^13^C NMR (CDCl_3_, 75 MHz): *δ* = 160.7 (C=O), 146.6, 145.0, 144.7, 144.6 (pyrazine-C), 60.6 (CH_2_, N–CH_2_), 51.2, 39.7, 28.3, 27.0 (CH_2_, CH_2_–CH_2_); HRESIMS *m/z* (pos): 266.3630 C_12_H_18_N_4_OS (calcd. 266.1201); Anal. Calcd. for C_12_H_18_N_4_OS: C, 54.11; H, 6.81; N, 21.03; S, 12.04. Found: C, 54.10; H, 6.82; N, 21.04; S, 12.04%.

### Biological activity experiments

TB bacillus strain was the standard strain H_37_Rv. The modified roche medium was used for bacteriostatic experiment, and the MH agar culture medium was used for the experiment of minimal inhibitory concentration (MIC). The standard strains were dissolved in sterile distilled water, and the bacteria liquid concentration was adjusted to 10^3^ CFU/mL. The 0.1 mL bacteria were accurately inoculated by pipetting gun on modified roche medium, then pyrazinamide derivative (0.1 mL, 25 µg/mL) was put into culture medium. Each compound was inoculated in five culture tubes, respectively. The culture tubes were put into an incubator with 5% CO_2_ at 36 °C for 20 days, and the wedding difference of coli growth was observed. Blank control experiment did not need to take drug, and directly inoculated with the microorganisms. For minimum bacteriostatic concentration experiment, various derivatives were mixed into different concentration solutions firstly, then they were moved into MH agar medium, respectively. After sterilisation, 0.1 mL standard H_37_Rv bacteria liquid with multi-point inoculator was inoculated in the agar plate surface. Vaccination was added into the incubator with 5% CO_2_ at 36 °C for 24 h, and the growth differences of n/med *Tuberculosis* bacili were observed.

## Results and discussion

### The design and synthesis of pyrazinamide derivatives

**Figure 2.**
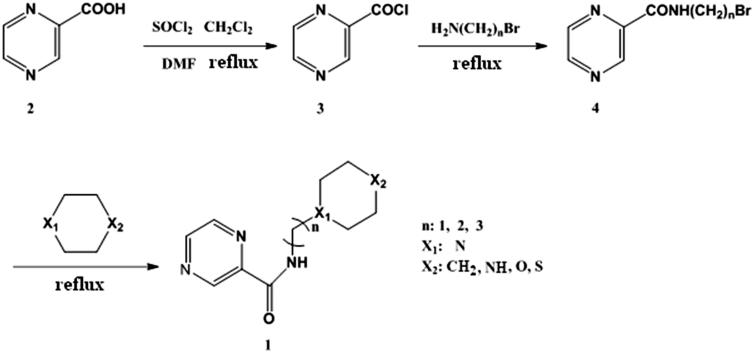
Synthetic route of pyrazinamide derivatives.

The pyrazinamide derivatives for anti-*Mycobacterium tuberculosis* bacilli were designed using the pyrazinamide as lead compound, which was modified with the alkyl chains/six-member rings and bioisosterism. In the structure transformation with alkyl chains, the number of carbon atom was 1, 2, and 3, respectively. In the structure transformation with six-member rings, the X_1_ = N; X_2_ = CH_2_, NH, O, S were selected, respectively. Through the optimisation of lead compound structure, 20 compounds were designed. Using pyrazinecarboxylic acid as the starting material, the pyrazinamide derivatives were synthesised by acylation, amidation, and alkylation, respectively. This synthetic route (Figure 2) was simple, and the yield was higher (79.6%–91.2%). It indicated that the length of alkyl chain did not obviously affect the reaction yield, but the six-member ring type more obviously influenced the yield. In general, the production rate with six-member ring (X_1_ = N and X_2_= O) was obviously higher than other combinations. In addition, it was showed that the chemical shift of –NH(CH_2_)*_n_*– changed from compound **4** to target product **1**. So, the synthesis was successful.

### Biological activities

Bioactive experiment process was based on standard H_37_Rv tuberculosis bacillus strains differences, the bacteriostatic experiment and minimum bacteriostatic concentration experiment were carried out, respectively. The modified roche medium was used for the bacteriostatic experiment, and the MH agar was used for the minimum bacteriostatic concentration experiment. Pure culture method and agar dilution method were used, respectively. The results of biological activity experiment are shown in Table 1. It showed that the length of alkyl chain and six-member ring kind had certain effect on the biological activity. For compound **1f** with the alkyl chain of carbon atom (*n* = 2) and X_1_ =N, X_2_ =O, its bacteriostatic effect was 99.2%, and the minimum bacteriostatic concentration was the lowest (8.0 µg/mL) among all compounds. Other bioactive compounds were ideal, the bacteriostatic rate was from 79.2% to 93.6%, and the minimum bacteriostatic concentration ranged from 10.2 µg/mL to 32.2 µg/mL. For the structure-activity, the alkyl chain and the six-member ring kind influenced the activity of pyrazinamide derivatives. When the carbon atom number of alkyl chain was 2, the molecular space structure was the best. In addition, the derivative with the six-member ring (X_1_=N, X_2_=O) had the biggest combining ability, so its biological activity was the best too. In the experiment, the pyrazinamide was used as a positive control.

**Table 1.** Biological activities of pyrazinamide derivatives (0.1 mL, 25 μg/mL).

Compounds	*n* X_1_ X_2_	Bacteriostatic rate (%)	MIC (μg/mL)
**1a**	1 N CH_2_	80.1	27.3
**1b**	1 N NH	86.7	23.4
**1c**	1 N S	88.4	20.2
**1d**	2 N CH_2_	85.9	15.4
**1e**	2 N NH	87.8	12.2
**1f**	2 N O	99.6	8.0
**1g**	2 N S	93.6	10.2
**1h**	3 N CH_2_	89.8	21.5
**1i**	3 N NH	90.7	18.3
**1j**	3 N O	92.0	16.2
**1k**	3 N S	91.3	17.1
Pyrazinamide	–	99.2	12.5

## Conclusion

A total of 11 pyrazinamide derivatives were designed and synthesised using pyrazinamide as the lead compound, which was modified with the alkyl chains/six-member rings and bioisosterism, respectively. The chemical structures of these derivatives were characterised by ^1^H NMR, ^13 ^C NMR, MS, and elemental analysis, respectively. These derivatives had been carried out the assay of biological activity. It showed that some derivatives had good function of resisting *M. tuberculosis*, high bacteriostatic rates, and low minimum bacteriostatic concentrations. In addition, the derivative **1f** had the best biological activity among all compounds, its antibacterial rate was 99.6%, and the minimum concentration of bacteriostasis was 8.0 µg/mL.
